# Intelligent Drug Delivery by Peptide-Based Dual-Function Micelles

**DOI:** 10.3390/ijms23179698

**Published:** 2022-08-26

**Authors:** Dong Wan, Yujun Liu, Xinhao Guo, Jianxin Zhang, Jie Pan

**Affiliations:** 1 School of Chemical Engineering and Technology, Tiangong University, Tianjin 300387, China; 2 School of Chemistry, Tiangong University, Tianjin 300387, China

**Keywords:** drug delivery, peptide, micelles

## Abstract

To endow the polymeric prodrug with smart properties through a safe and simple method, matrix metalloproteinase (MMPs) responsive peptide GPLGVRGDG was introduced into the block copolymer to prepare TPGS_3350_-GPLGVRGDG-DOX&DOX micelles, where TPGS_3350_ is D-α-tocopheryl polyethylene glycol 3350 succinate. During the doxorubicin delivery, the cleavage of the peptide chain triggers de-PEGylation, and the remaining VRGDG sequence was retained on the surface of the micelles, which can act as a ligand to facilitate cell uptake. Moreover, the cytotoxicity of TPGS_3350_-GPLGVRGDG-DOX&DOX micelles against 4T1 cells was significantly improved, compared with TPGS_3350_-GPLGVRG-DOX&DOX micelles and TPGS_3350_-DOX&DOX micelles. During in vivo studies, TPGS_3350_-GPLGVRGDG-DOX&DOX micelles exhibited good anticancer efficacy with long circulation in the body and more efficient accumulation at the tumor site. Therefore, TPGS_3350_-GPLGVRGDG-DOX&DOX micelles have improved antitumor activity and reduced toxic side effects. This work opens new potential for exploring the strategy of drug delivery in clinical applications.

## 1. Introduction

Currently, as the incidence of malignant tumor rises, cancer is a leading cause of death worldwide [1,2,3]. Doxorubicin (DOX) is commonly used in treatment of breast, lung, gastric, and thyroid cancers [4,5]. Although DOX is widely used in clinical practice, this conventional anticancer drug generally has some disadvantages, such as short in vivo half-life, poor selectivity, and high toxic side effects [6]. Therefore, a delivery system that effectively delivers drugs to cancer cells is very important for the clinical efficacy of anticancer drugs.

Many methodologies were studied to improve the delivery efficiency of anticancer drugs, among which the preparation of polymeric micelle drugs is a promising strategy for achieving this goal [7,8,9,10,11,12,13,14]. Compared with conventional anticancer drugs, prodrug micelles prepared by nanomedicine have some advantages, such as, improving the pharmacokinetics and accumulation of drugs at the tumor site, increasing the bioavailability of drugs, and reducing the side effects of drug toxicity on the organism [15,16,17]. To prolong the circulation time of drug carriers in vivo, polyethylene glycol (PEG) modification and other modifications are often exploited in the designing of drug carriers, which could be passively enriched at the tumor site based on the EPR effect [18,19]. It was reported that some of the confirmed and documented side effects of PEG include PEG allergy, digestive issues, and electrolyte imbalances [20]. Although PEG has these side effects, it is the most used biomaterial and the gold standard for stealth polymers in drug delivery [21].

The liposomal formulation of DOX (Doxil^®^), for example, was the first nanomedicine to be approved by the US Food and Drug Administration (FDA), and it has a longer circulation time and lower toxicity than DOX [22]. However, studies found that conventional nanocarriers could not significantly improve the efficacy of chemotherapy due to their large size, slow drug release, and lack of active targeting. In addition, PEG modification was not conducive to the internalization of drugs, leading to a poor effect of anticancer [23,24]. To solve the above problems, researchers designed a variety of intelligent polymeric drug delivery systems to further improve the therapeutic effect [25,26,27,28]. Matrix metalloproteinases play an important role in tumor growth and metastasis [29,30]. Collagenase type IV, including MMP-2 and MMP-9, is one of these vital types [31]. It was highly expressed in almost all tumor tissues and secreted by tumor cells, which had obvious advantages in comparison with intracellular environment [32]. Recently, MMPs were widely used as an effective inducer of tumor imaging and drug release. However, the preparation of a delivery system with a well-defined structure remains a great challenge.

Introduction of a peptide block as a stimuli-responsive linkage between PEG and DOX is an important approach to endow nanomaterials with multi-functional properties for better therapeutic results [33,34]. Peptides and their derivatives attracted the attention due to excellent biocompatibility, diverse bioactivity, potential biodegradability, specific recognition, and ease of chemical modification [35,36]. At the same time, peptides are rich in monomeric amino acids and have side chains that can be modified with functional groups, allowing them to respond to a variety of stimuli, making them easy to integrate into conventional cancer treatment systems [37,38]. Therefore, many researchers developed peptides and combined them with imaging molecules or therapeutic drugs for the diagnosis and treatment of tumors [39]. Nanomaterials of peptides have great potential for biomedical applications, especially in the field of cancer therapy.

**Scheme 1:**
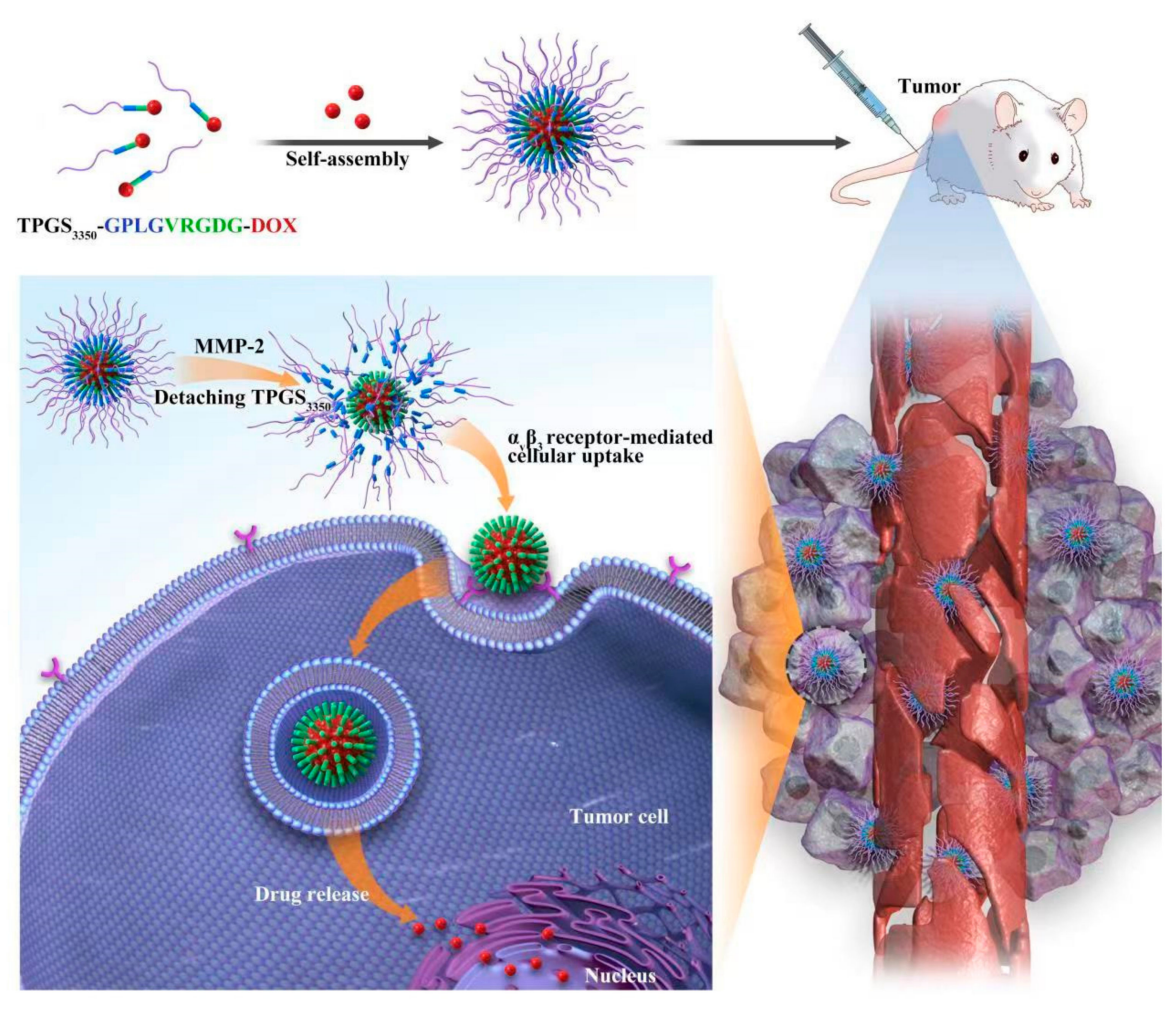
The structure and drug delivery of the peptide-based dual-function micelles.

In this work, DOX-loaded TPGS_3350_-GPLGVRGDG-DOX micelles (P1) were prepared (Scheme 1). TPGS is D-α-tocopheryl polyethylene glycol 1000 succinate, which is a fabricated esterification of vitamin E succinate with polyethylene glycol 1000. TPGS is an ideal biomaterial for drug delivery, containing an amphiphilic structure with a 13.2 hydrophilic/lipophilic balance (HLB) value and 0.02% w/w low critical micelle concentration (CMC) [14]. TPGS_3350_ is D-α-tocopheryl polyethylene glycol 3350 succinate, which can reduce the nonspecific uptake by cells to prolong the circulation time of the nanocarriers in the blood [29]. P1 responds to the microenvironment of the tumor to overcome the bio-barriers during DOX delivery in vivo. Under normal physiological conditions, the outer layer of TPGS_3350_ with PEG_3350_ prolonged the circulation time of micelles in blood [40]. The peptide GPLGVRGDG (Gly-Pro-Leu-Gly-Val-Arg-Gly-Asp-Gly) introduced in the structure was sensitive to MMP-2 enzymes. The high expression of MMP-2 enzyme in the tumor tissues cleaved the peptide GPLGVRG between G and V and removed the TPGS_3350_ outer layer, making it easier to penetrate the tumor site [41]. At the same time, the exposed VRGDG ligand actively targeted to α_v_β_3_ receptor was overexpressed on the surface of the tumor cells [42,43]. Thus, these micelles achieved excellent efficiency of drug delivery, high toxicity to cancer cells, and low side effects on normal tissues.

## 2. Results and Discussion

### 2.1. Synthesis and Characterzation of Polymeric Prodrugs

**Figure 1:**
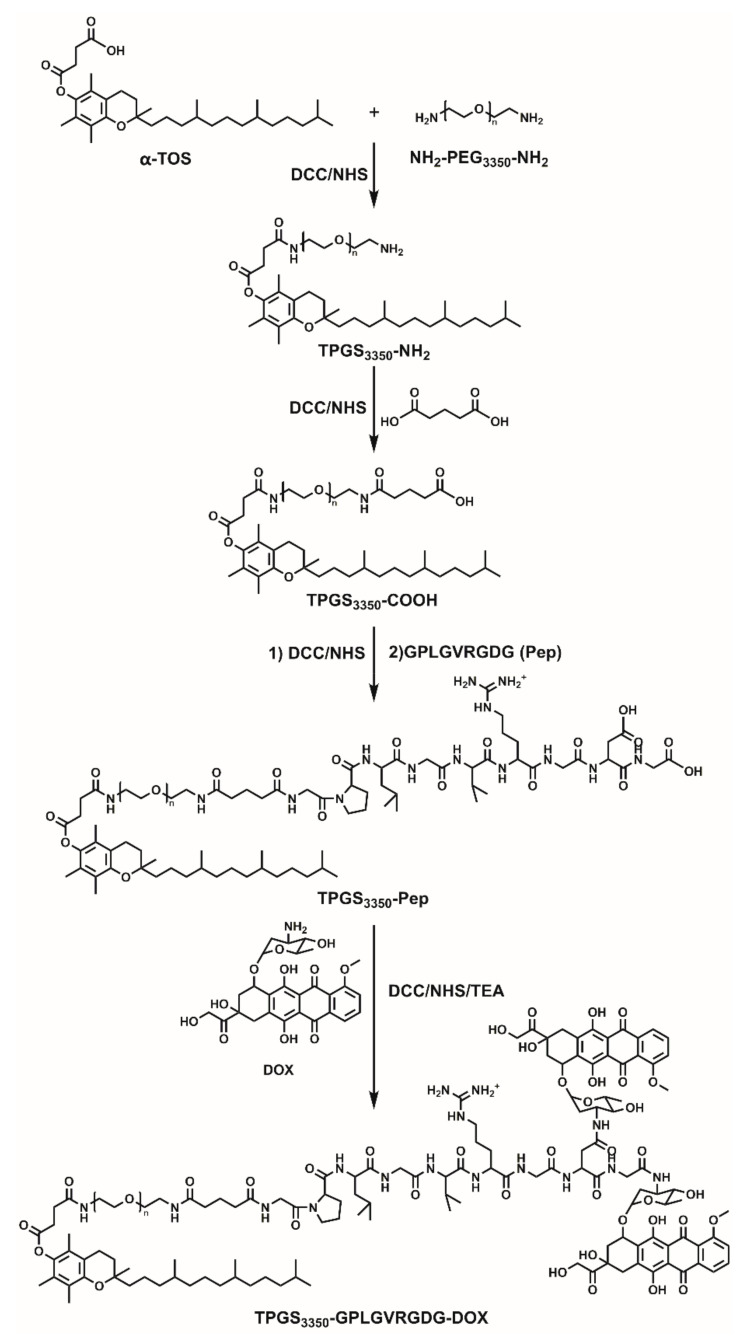
Synthetic route of TPGS_3350_-GPLGVRGDG-DOX.

Polymeric prodrug TPGS_3350_-GPLGVRGDG-DOX was synthesized, which is shown in Figure 1. The structure was confirmed by ^1^H NMR (Figure S1). In the ^1^H NMR spectrum, the peaks at 1.89–1.91 ppm were assigned to -CO-CH_2_- on the peptide. The peaks at 3.41–3.57 ppm were attributed to -CH_2_CH_2_O- on PEG. The peaks at 7.75–8.10 ppm belonged to the protons on an aromatic ring in DOX, which verified the successful synthesis of TPGS_3350_-GPLGVRGDG-DOX.

### 2.2. Characterization of Micelles

**Figure 2:**
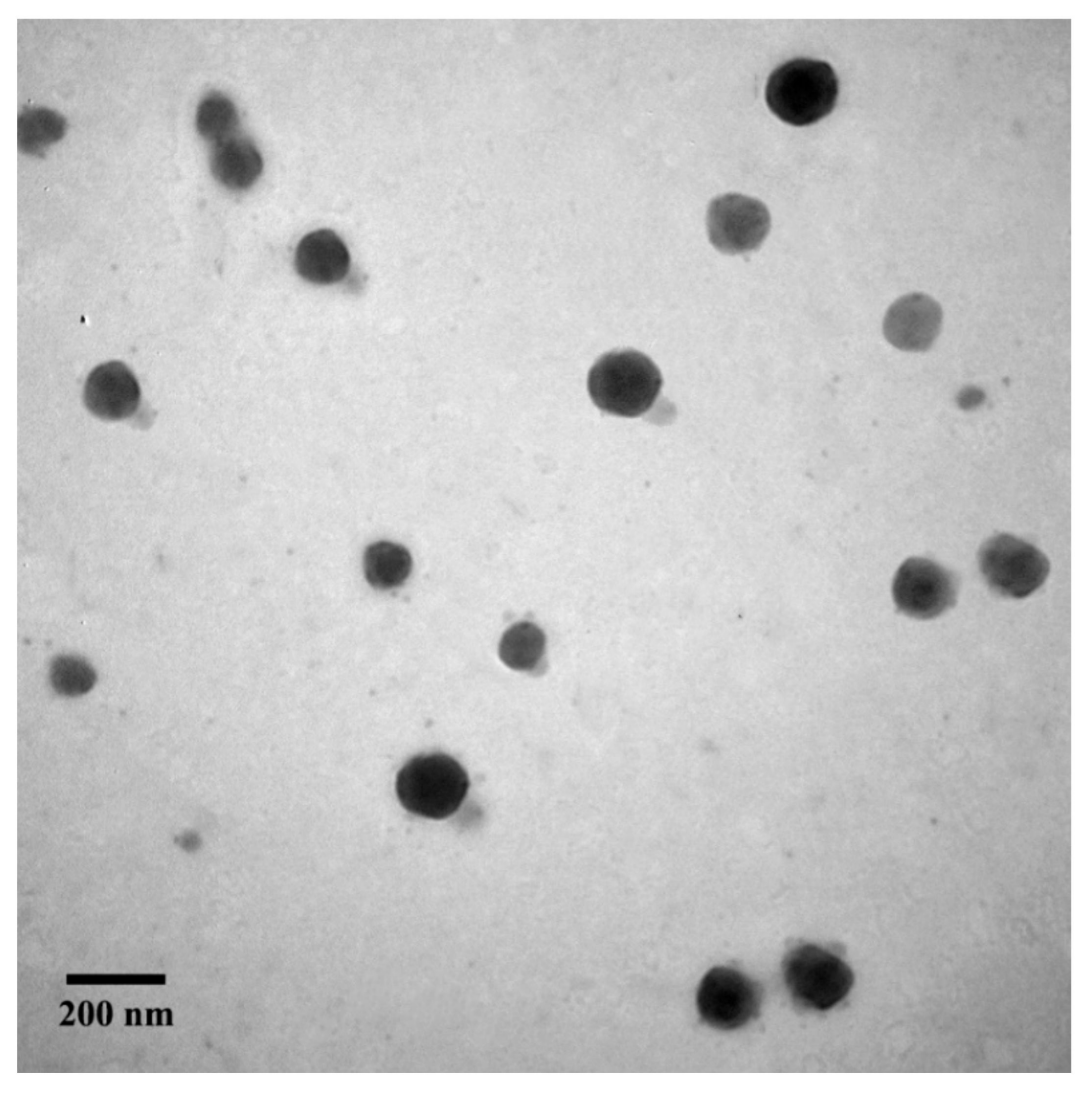
TEM image of P1.

**Figure 3:**
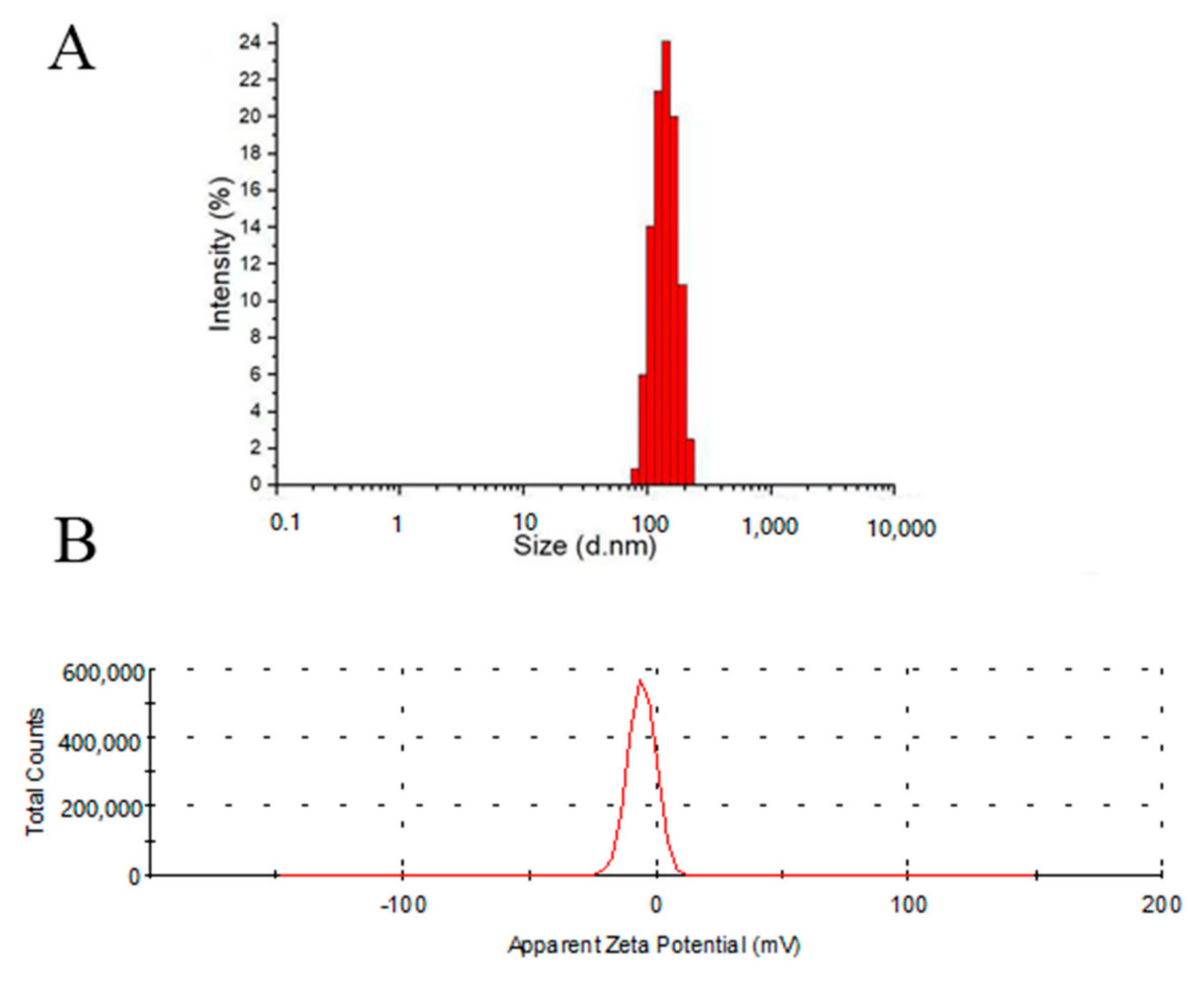
Size distribution (**A**) and zeta potential (**B**) of P1.

The micelle with enzyme response and active targeting prepared with TPGS_3350_-GPLGVRGDG-DOX prodrug was P1, the micelle with enzyme response fabricated by TPGS_3350_-GPLGVRG-DOX prodrug (GPLGVRG is Gly-Pro-Leu-Gly-Val-Arg-Gly) was P2, and the micelles from TPGS_3350_-DOX prodrug were P3. The morphologies of P1 were collected by transmission electron microscope (TEM) (Figure 2), from which it could be found that P1 exhibited a relatively homogeneous spherical morphology with an average size of about 130 nm. The dynamic light scattering (DLS) data in Figure 3A indicates that the particle size of P1 was 140.6 ± 3.2 nm, which was slightly larger than that in TEM. It was reported that micelles with a particle size less than 200 nm could achieve long circulation in vivo and accumulate more readily in tumor sites via EPR effects [16]. The zeta potential of P1 was –4.97 mV (Figure 3B), which indicates that P1 would be stable in aqueous solutions. Additionally, the critical micelle concentration (CMC) of P1 was determined to be 5.71 × 10^−3^ mg/mL, which facilitated the stability in blood circulation. The drug loading contents (DLC) of P1, P2, and P3 were 12.45%, 10.63%, and 12.07%, and the drug encapsulation efficiency (DEE) were 61.49%, 53.87%, and 59.51%, respectively. The DLC and DEE values for P1, P2 and P3 are similar.

### 2.3. In Vitro Drug Release

**Figure 4:**
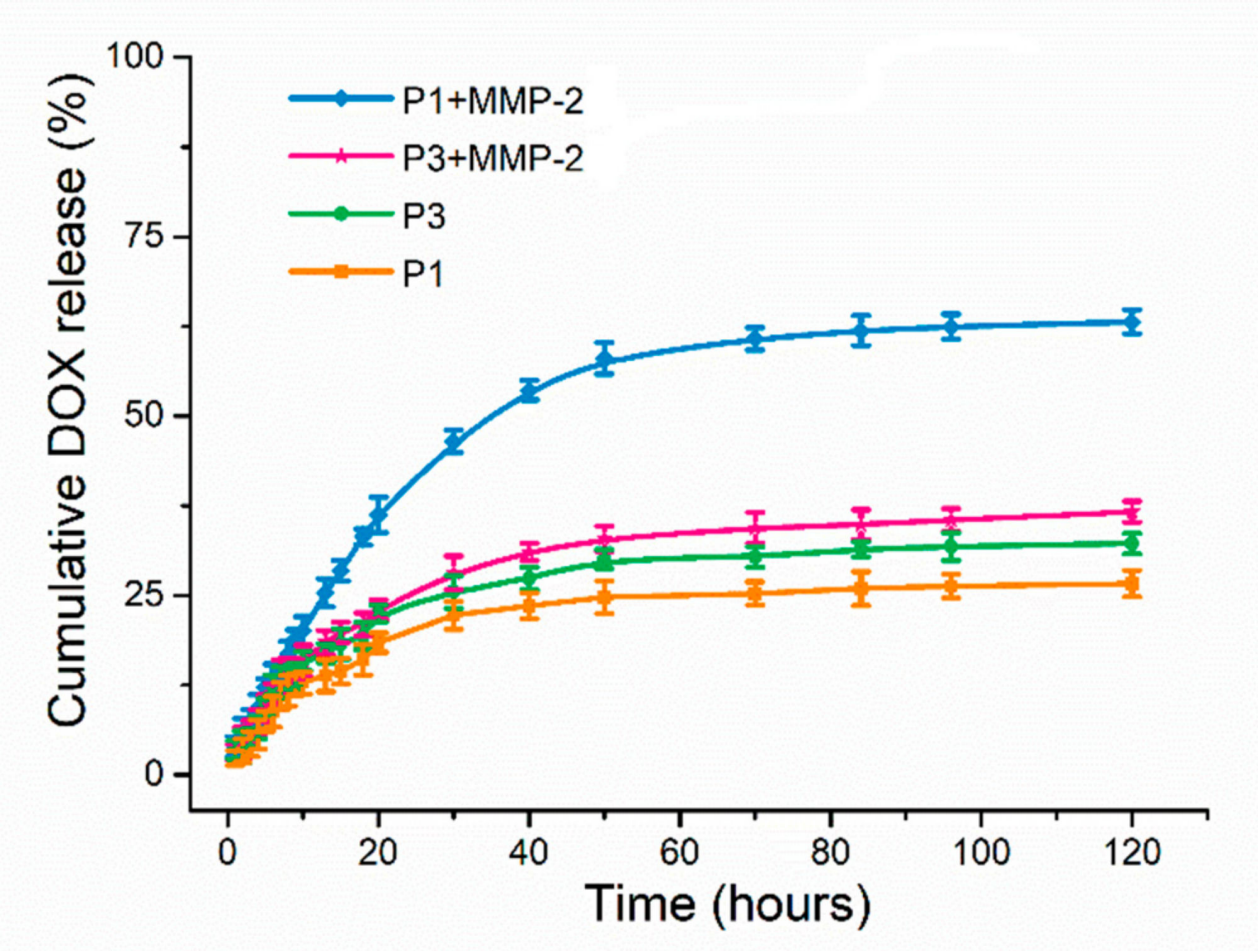
In vitro release of DOX from P1 and P3.

Figure 4 illustrates the cumulative in vitro DOX release of P1 and P3 with or without MMP-2 enzyme in 120 h at 37 °C in Phosphate-Buffered Saline (PBS). After incubation with the MMP-2 enzyme for 120 h, 63.13% of DOX in P1 was released with a significantly higher cumulative release than the other three groups. P1 had MMP-2 enzyme-responsive properties and achieved responsive breakage of peptide fragments, resulting in an increasing release of DOX. In contrast, only 36.63% of DOX in P3 was released in the presence of MMP-2 after 120 h. Furthermore, approximately 30% of DOX was released in the other two groups without MMP-2. It was confirmed that P1 was stable in the normal physiological environment of the human body and had the ability to bind DOX without releasing the drug prematurely.

### 2.4. In Vitro MMP-2 Enzyme Responsiveness

**Figure 5:**
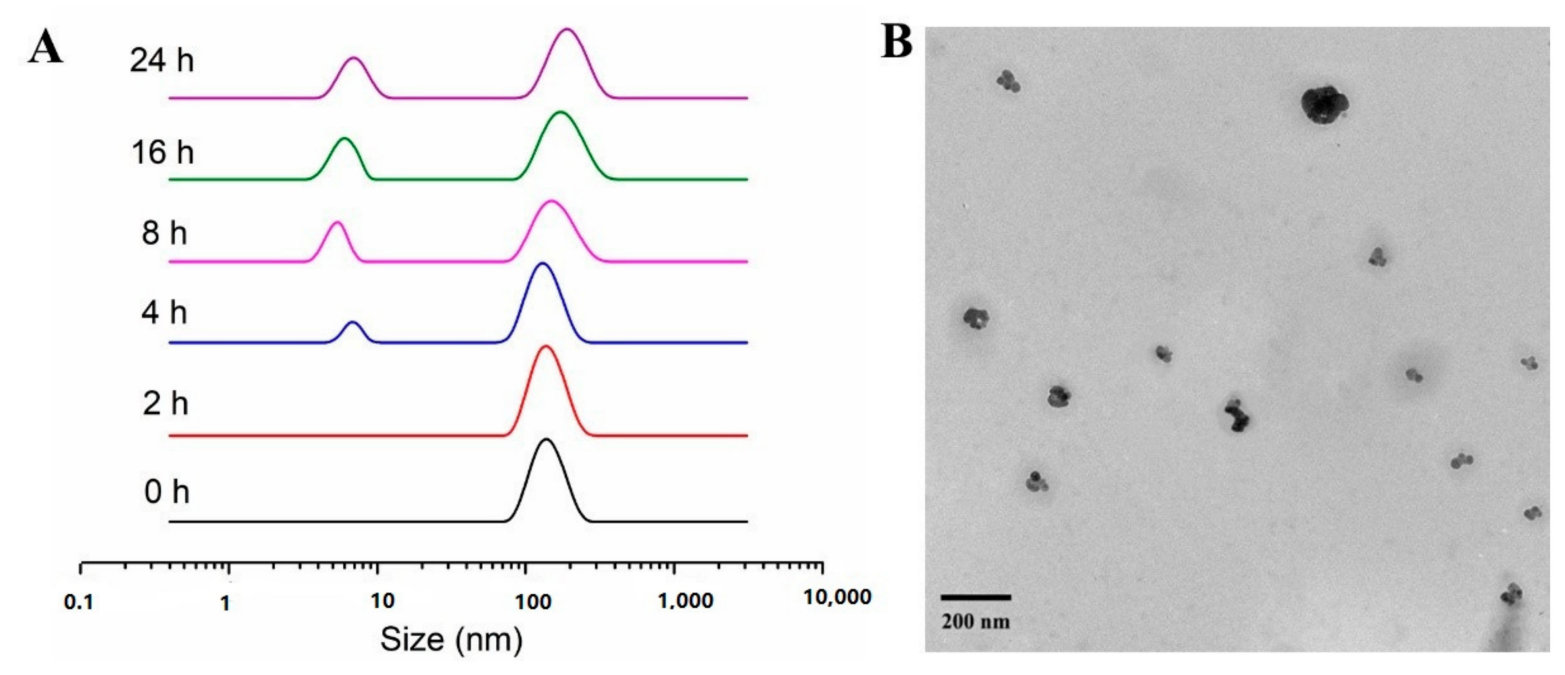
Size distribution (**A**), and TEM image (**B**) of P1 after treatment of MMP-2.

Figure 5A demonstrates that the nanoparticle size of P1 was obviously decreased after treatment with the MMP-2 enzyme. The morphological changes of P1 in an environment containing MMP-2 enzymes were further verified by TEM (Figure 5B). These results indicated that the enzyme-responsive peptide in P1 was structurally altered after cleavage by MMP-2 enzyme and the surface modification of TPGS_3350_ was removed.

### 2.5. In Vitro Cytotoxicity

**Figure 6:**
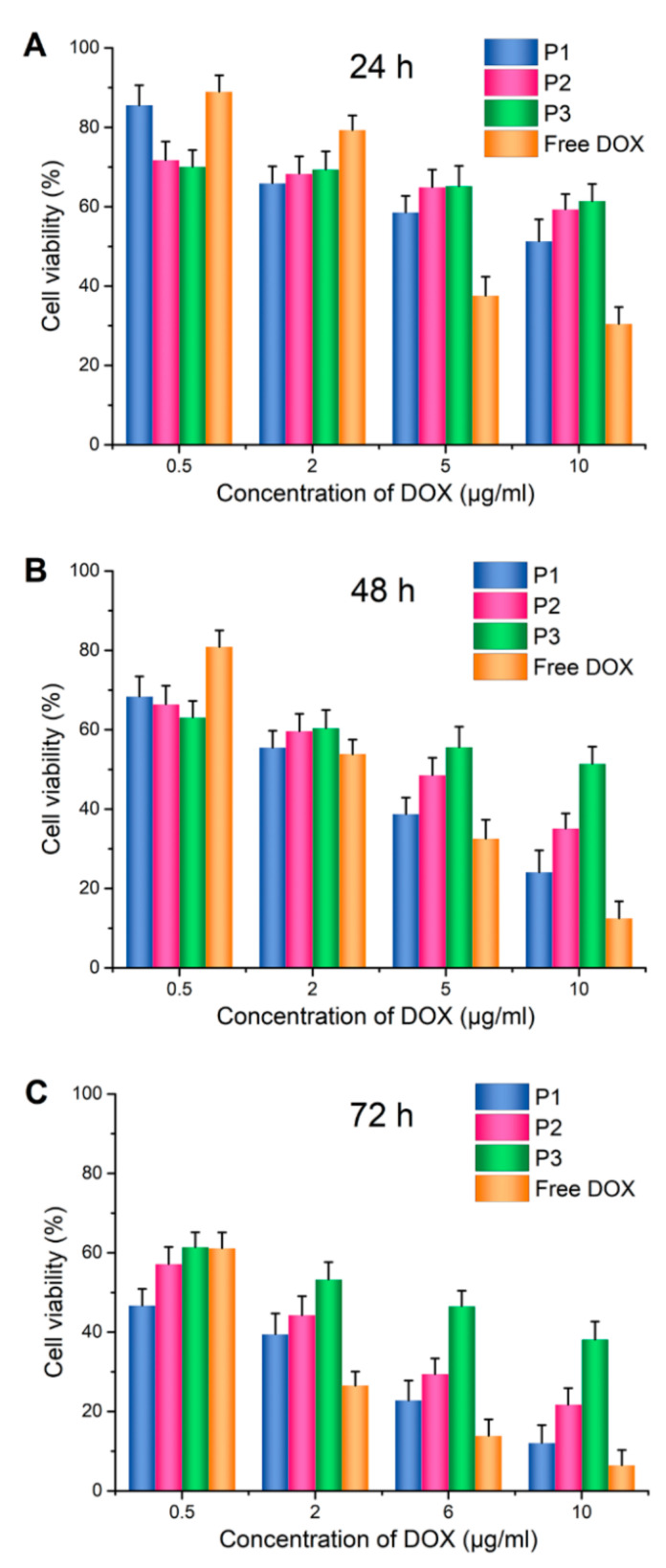
Cell viability of 4T1 cells after incubation with P1, P2, P3, and free DOX for 24 h (**A**), 48 h (**B**), and 72 h (**C**).

To determine the cytotoxicity of the micelles, 4T1 cells were incubated at DOX, P1, P2, and P3 with different drug concentrations for 24 h, 48 h, and 72 h, respectively. It could be seen in Figure 6 that in all formulations, the longer the incubation time was, the lower the survival rate of 4T1 cells. When both time and drug concentration reached a certain level, P1 presented stronger cytotoxicity to 4T1 cells than those of P2 and P3. It should be noted that when the DOX concentration was 10 μg/mL, the survival rate of 4T1 cells after 72 h was only 12.08% for the P1, 21.71% for P2, and 38.13% for P3. In addition, the half-maximum inhibitory concentration (IC_50_) value of DOX in P1 was 0.32 μg/mL, which was much lower than the value of P2 (1.29 μg/mL) and P3 (4.56 μg/mL). The increased uptake of P1 by tumor cells may be due to the removal of TPGS_3350_ under the action of MMP-2 to expose the active targeting peptide of VRGDG, which can bind to the overexpressed integrin receptor α_v_β_3_ on the surface of the tumor cells, entering cells through receptor-mediated endocytosis, and rapidly releasing DOX in cells.

### 2.6. In Vitro Cellular Uptake

**Figure 7:**
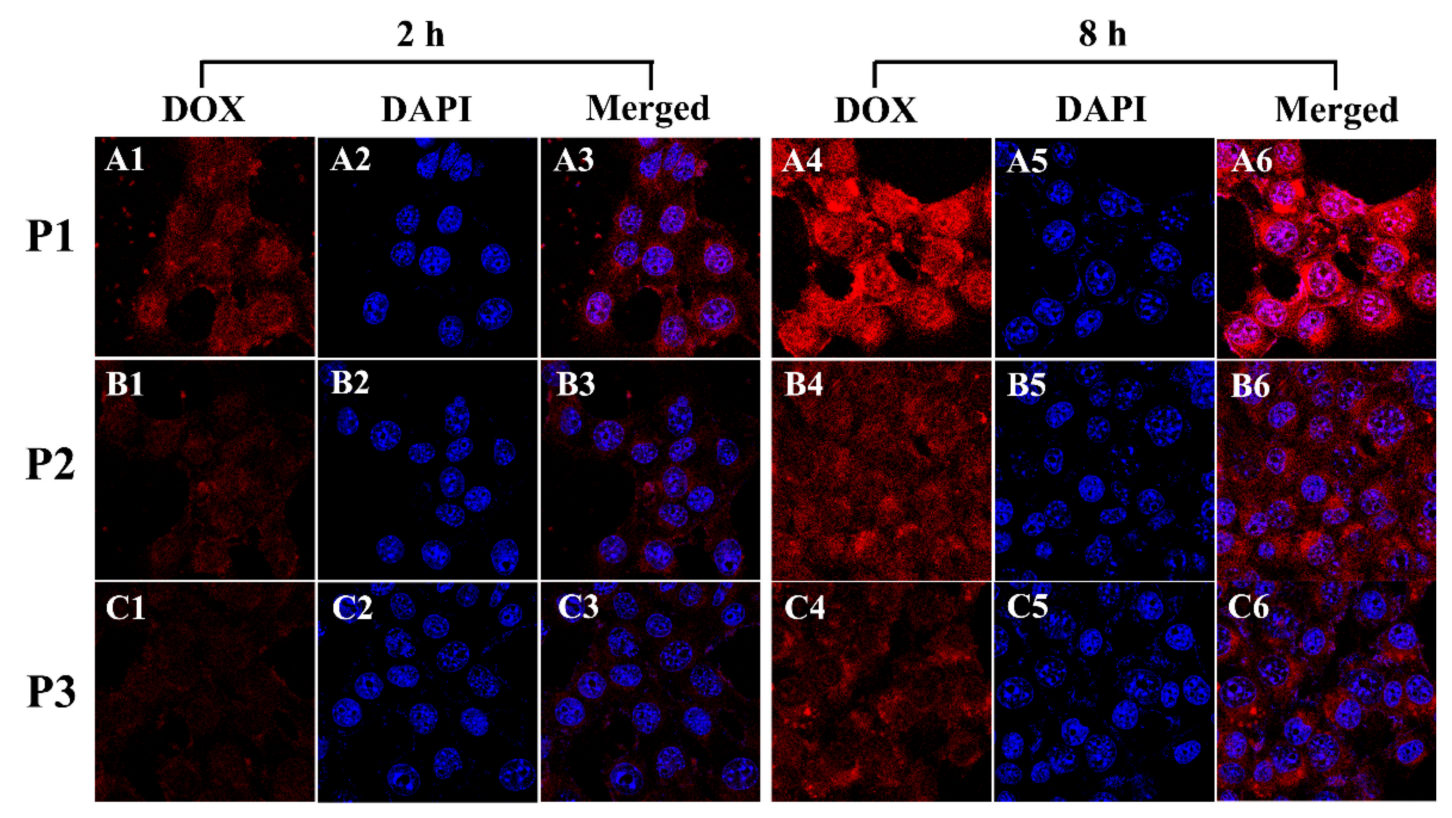
CLSM images of 4T1 cells incubated with P1 (**A1**–**A6**), P2 (**B1**–**B6**), P3 (**C1**–**C6**) for 2 h and 8 h, respectively.

The cellular uptake of 4T1 cells was recorded by using Confocal Laser Scanning Microscopy (CLSM). In Figure 7, there was a strong red fluorescence in the cytoplasm, surrounding the nucleus (blue) after P1 was incubated with 4T1 cells for 2 h. This phenomenon indicates that the DOX-loaded micelles were effectively taken up by the tumor cells. Furthermore, a stronger red fluorescence of P1 was observed, compared to P2 and P3 after 2 h and 8 h incubation with 4T1 cells, which reveals that P1 exhibited efficient cellular internalization. Consequently, the size of P1 was reduced by the effect of the MMP-2 enzyme to remove the outer layer of PEG. At the same time, the targeting peptide bound to the α_v_β_3_ integrin receptor was overexpressed on the surface of the tumor cells, leading to more P1 entering into cells via the receptor-mediated endocytosis pathway.

### 2.7. In Vivo Antitumor Efficacy

**Figure 8:**
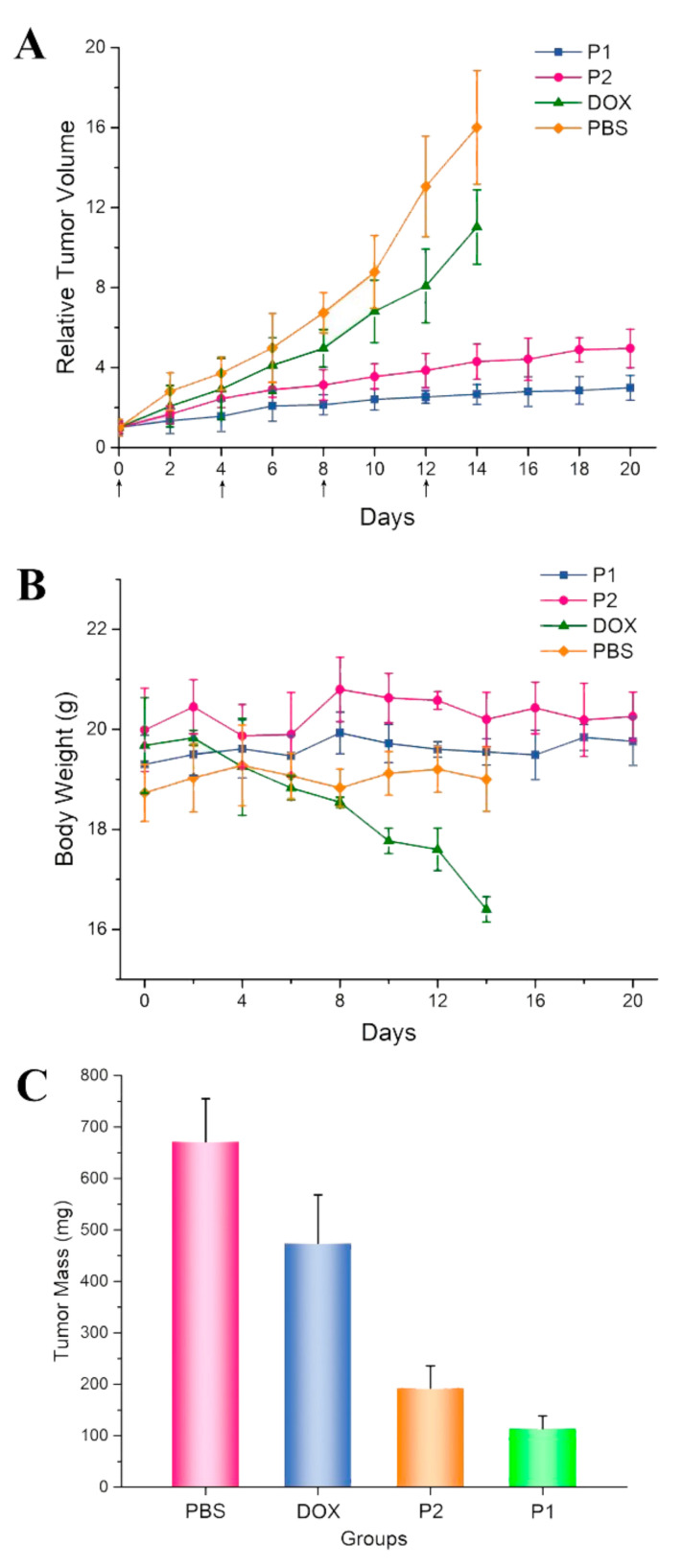
(**A**) Changes in tumor volume after intravenous injection of P1, P2, DOX, and PBS (Small arrows refer to the time of administration in mice). (**B**) The changes of body weight. (**C**) Final tumor weights.

The in vivo anticancer efficacy of micelles was also evaluated with a 4T1 mouse breast cancer cell xenografted model. The tumor volume of the mice in the PBS group increased rapidly during the treatment, while the tumor volume of the mice in the DOX group increased slower than that of the PBS group. However, the body weight decreased significantly. According to the growth state, the mice in the PBS group and the DOX group were sacrificed on the 14th day. Figure 8A demonstrates the changes in the tumor volume of these mice injected with different formulations. After 14 days, the tumor volume increased 16.01-fold in the PBS group and 11.03-fold in the DOX group. Clearly, P1 expressed high tumor inhibition with slight 2.99-fold growth of the tumor volume in comparison with P2 (4.96-fold) at the end of the experiment. These results confirmed that P1 exhibited a better anticancer effect. Furthermore, Figure 8B illustrates the changes in the body weight of the mice in each group. The body weight in the P1 group did not change significantly during treatment, implying that P1 demonstrated outstanding biosafety. In additions, 0.11 g, 0.19 g, 0.47 g, and 0.67 g of tumor weight were found for P1, P2, DOX, and free DOX, respectively. The final tumor weights from each group in Figure 8C further confirmed that P1 exhibited the best anticancer efficiency.

**Figure 9:**
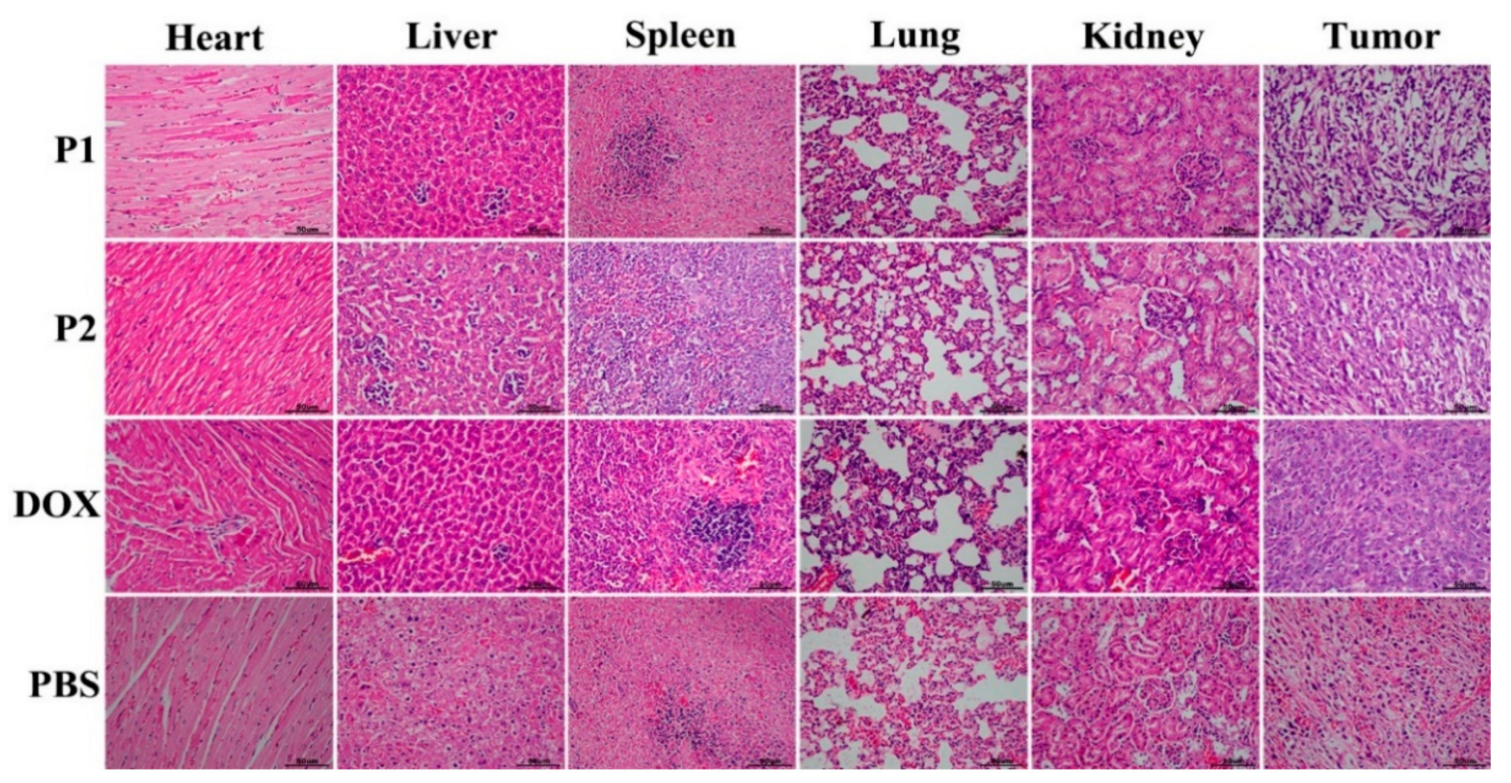
H&E staining of tumor tissues and hearts, livers, spleens, lungs, kidneys after treatment with various formulations (Scale bar: 50 μm).

To further evaluate antitumor effect of micelles, tumors and major organs were stained with H&E after being treated with different formulations. As shown in Figure 9, tumor tissues illustrate varying degrees of apoptosis. The histological H&E staining results indicated that the suppression effect of P1 was much better than that of other groups. Specifically, the percentage of apoptotic cells in tumors treated with P1 was much higher than that in tumors treated with P2, DOX, and PBS. Meanwhile, the cell morphology in the rest of the major organ sections was normal. Therefore, P1 has an improved antitumor efficacy and reduced toxic effects on other normal tissues.

## 3. Materials and Methods

### 3.1. Materials

D-α-tocopheryl succinate was obtained from Aladdin Industrial Corporation. Sinopharm Chemical Reagent Co., Ltd. (Shanghai, China) provided N, N’-dicyclohexylcarbodiimide (DCC), N-hydroxy succinimide (NHS), and N-(3-Dimethylaminopropyl)-N′-ethyl carbodiimide hydrochloride (EDC). Polyethylene glycol diamine 3350 was purchased from Chemgen Pharma Co. Ltd. N, N-Dimethylformamide (DMF), dichloromethane (DCM), methanol, ether, and dimethyl sulfoxide (DMSO) were all from Tianjin Kemiou Chemical Reagent Co. Ltd. Trifluoroacetic acid, Triisopropylsilane, N-methyl morpholine, and 1, 8-Diazabicyclo [5.4.0] under-7-ene (DBU) were purchased from J&K Technology Co., Ltd. (Hong Kong) Beijing Huafeng United Technology Co., Ltd. (Beijing, China) offered Doxorubicin (DOX). The Chinese Academy of Medical Sciences supplied mouse melanoma cells (4T1). Cell counting kit-8 (CCK-8) assays were bought from Shanghai Beyotime Institute of Biotechnology (Shanghai, China).

### 3.2. Synthesis of Polymer

According to the standard solid-phase peptide synthesis (SPPS) technology, the MMP-2 cleavable tumor-active targeting peptide (GPLGVRGDG) and its control sample (GPLGVRG) with MMP-2 cleavable peptide were prepared by Fmoc-coupling chemistry [44].

TPGS_3350_-COOH: D-α-tocopheryl succinate (1 mmol), PEG _3350_ (1.2 mmol), NHS (1 mmol), and DCC (1 mmol) were dissolved in dichloromethane in a round bottom flask, and 20 μL of triethylamine (TEA) was added dropwise, and reacted for 48 h in nitrogen after being completely dissolved. The byproduct was precipitated with cold ether, and the product TPGS_3350_-COOH was obtained after dialysis with distilled water for 48 h and freeze-drying.

TPGS_3350_-GPLGVRGDG: TPGS3350-COOH, DCC, and NHS were added to DMF in a stoichiometric molar ratio of (1:1.2:1.2) and stirred in a round bottom flask under nitrogen protection for 24 h. The peptide (GPLGVRGDG) (1.2 mmol) was dissolved in 5 mL DMF and then mixed into the above solution and stirred for 24 h. The resulting solution was dialyzed against DMF and distilled water, and freeze-dried to obtain a white solid.

TPGS_3350_-GPLGVRGDG-DOX: TPGS_3350_-GPLGVRGDG (1 mmol) was dissolved in 5 mL DMF along with DCC (1.2 mmol), NHS (1.2 mmol), and DOX (1.2 mmol) and stirred at 60 °C for 24 h. Finally, TPGS_3350_-GPLGVRGDG-DOX powder was obtained after dialysis and freeze-drying.

The copolymers TPGS_3350_-GPLGVRG-DOX and TPGS_3350_-DOX were synthesized as experimental controls concerning the method used to prepare TPGS_3350_-GPLGVRGDG-DOX.

### 3.3. Characterizaton of TPGS_3350_-GPLGVRGDG-DOX

The chemical structure of the prodrug was characterized by a ^1^H NMR spectrometer (Bruker ARX 400 MHz spectrometer, Ettlingen, Germany) with DMSO-d_6_ as the solvent.

### 3.4. Fabricaton and Characterizaton of Micelles

The DOX-loaded TPGS_3350_-GPLGVRGDG-DOX micelles were fabricated by thin-film hydration method. Briefly, 10 mg TPGS_3350_-GPLGVRGDG-DOX and 1 mg DOX were dissolved in a round bottom flask containing 1.5 mL DCM, a thin film that appeared at the bottom of the flask after rotary evaporation. Then, 2 mL deionized water was added and stirred for 30 min. The precipitate was removed by filtration through a 0.45 μm filter membrane, and the final product P1 was collected by freeze-drying. P2 and P3 were prepared by using the same method as P1.

### 3.5. Characterization of P1

The particle size distribution and zeta potential of P1 were measured by a laser particle size analyzer (Malvern Matersizer 2000, Westborough, MA, USA) at 25 °C. The morphology of the P1 was investigated by transmission electron microscope (Hitachi H7650, Tokyo, Japan). The critical micelle concentration was determined with a fluorescent probe using pyrene. DLC and DEE of micelles were measured with UV-vis. DOX was dissolved in DMSO to prepare a series of concentration solutions, and the UV absorption intensity of DOX at different concentrations was scanned by an enzyme calibrator (Perkin Elmer Victor III, Elkin, NC, USA) to obtain the UV absorption curves of different concentrations of DOX. The UV absorption was also measured by dissolving P1 (2 mg) in DMSO (10 mL). The relevant formulas are as follows.

(1) $$\mathrm{DLC}(\%)=\frac{\mathrm{weight}\;\mathrm{of}\;\mathrm{the}\;\mathrm{DOX}\;\mathrm{in}\;\mathrm{micelles}}{\mathrm{weight}\;\mathrm{of}\;\mathrm{micelles}}\times 100\%,$$

(2) $$\mathrm{DEE}(\%)=\frac{\mathrm{weight}\;\mathrm{of}\;\mathrm{the}\;\mathrm{DOX}\;\mathrm{in}\;\mathrm{micelles}}{\mathrm{weight}\;\mathrm{of}\;\mathrm{feeding}\;\mathrm{DOX}}\times 100\%.$$

### 3.6. Drug Release

The drug release from micelles was characterized by a dialysis method. In brief, P1 and P3 solution were transferred into a dialysis bag (MWCO = 1000 Da) in PBS with or without collagenase type IV at pH 7.4, respectively. The samples were shaken at 37 °C for 1, 2, 3, 4, 5, 6, 8, 10, 12, 20, 28, 36, 48, 60, 72, 96, 120 h, and external buffer was obtained to analyze the content of DOX, determined with a fluorescence detector at 480 excitation wavelength and 586 nm emission wavelength.

### 3.7. In Vitro MMP-2 Enzyme Responsiveness

The enzymatic reaction behavior of MMP-2 was verified by adding appropriate amounts of type IV collagenase to P1. The change in the particle size of P1 was measured by a laser particle size analyzer and the particle size morphology was observed by TEM after the enzymatic reaction.

### 3.8. In Vitro Cytotoxcity

To verify the effectiveness of P1, the cytotoxicity of different drug concentrations and dosage forms on 4T1 cells (mouse breast cancer cells) was measured with CCK-8 assays. A medium containing P1, P2, P3, and free DOX was added to each well. Then, 24 h, 48 h, and 72 h later, the absorbance of each well was measured at 450 nm using a microplate reader (Perkin-Elmer Victor III, Elkin, NC, USA).

### 3.9. In Vitro Cellular Uptake

A confocal laser scanning microscope (CLSM, Leica TCSNT1, Leverkusen, Germany) was used to observe the cell uptake. After 24 h of 4T1 cell culture, P1, P2, and free DOX were added and incubated for 30 min, 2 h, and 6 h. Nuclei were stained with DAPI.

### 3.10. In Vivo Antititumor Efficacy

All animal experiments were performed complying with the guidelines for the care and use of laboratory animals of the Peking University Animal Study Committee’s requirements. Experimental procedures were carried out by a protocol approved for institutional animal care. A total of 24 BALB/c female mice (5–6 weeks) were used in this study. The PBS suspension containing 2.5 × 10^4^ 4T1 cells was injected subcutaneously into female BALB/c mice. When the tumor volume increased to 50–100 mm^3^, different drugs (PBS/DOX/P1/P2) were injected via the tail vein of mice on days 0, 4, 8, and 12. The body weight and tumor volume of each mouse were measured at regular intervals to evaluate the antitumor effects and toxicity of different drugs. All mice were sacrificed on the 20th day. One mouse from each group was randomly selected, and its major organs and tumor tissues were collected, then Hematoxylin and Eosin (H&E) staining were utilized to evaluate the degree of apoptosis in mice.

## 4. Conclusions

In conclusion, novel peptide-based multifunctional micelles were successfully constructed for targeting tumor cells and efficient drug delivery. The average particle size of the micelles was 140.6 nm, and the surface potential was −4.97 mV, which was conducive to their long circulation in blood and aggregation at the tumor site through the EPR effect. The curve of in vitro drug release also showed that when P1 was treated with the MMP-2 enzyme, the cumulative drug release was the largest compared with those from the control groups, indicating that P1 is responsive to MMP2 enzyme. In vitro and in vivo experiments also revealed that P1 can actively target tumor cells to achieve significant therapeutic effects. Thus, the peptide-based drug delivery system designed in this work provides a new strategy for the development of anticancer formulations and shows potential for clinical application for tumor treatment.

## Data Availability

Numerical raw data can be provided on request. All other data are presented in the paper.

## Supplementary Materials

The supplementary materials can be downloaded at: https://www.mdpi.com/article/10.3390/ijms23179698/s1.

## Abbreviations

MMPs	matrix metalloproteinase
DOX	doxorubicin
TPGS_3350_	D-α-tocopherol polyethylene glycol 3350 succinate
GPLGVRGDG	Gly-Pro-Leu-Gly-Val-Arg-Gly-Asp-Gly
GPLGVRG	Gly-Pro-Leu-Gly-Val-Arg-Gly
VRGDG	Val-Arg-Gly-Asp-Gly
PEG	polyethylene glycol
EPR	enhanced permeability and retention
FDA	US Food and Drug Administration
MMP	matrix metalloproteinase
HLB	hydrophilic/lipophilic balance
CMC	critical micelle concentration
RGD	Arg-Gly-Asp
PBS	Phosphate-Buffered Saline
P1	TPGS_3350_-GPLGVRGDG-DOX & DOX
P2	TPGS_3350_-GPLGVRG-DOX & DOX
P3	TPGS_3350_-DOX & DOX
