# Supplementary material for: Intelligent Drug Delivery by Peptide-Based Dual-Function Micelles
Source: Int J Mol Sci. 2022 Aug 26;23(17):9698. doi: 10.3390/ijms23179698 (PMC9456463; doi:10.3390/ijms23179698)
Supplement: Supplementary file 1 [file ijms-23-09698-s001.zip › ijms-1827853-supplementary.pdf]

International Journal of
Molecular Sciences

Article

Intelligent Drug Delivery by Peptide-Based Dual-Function Micelles

Dong Wan [1], Yujun Liu [1], Xinhao Guo [1], Jianxin Zhang [2,*] and Jie Pan [1,*]

Supplementary materials

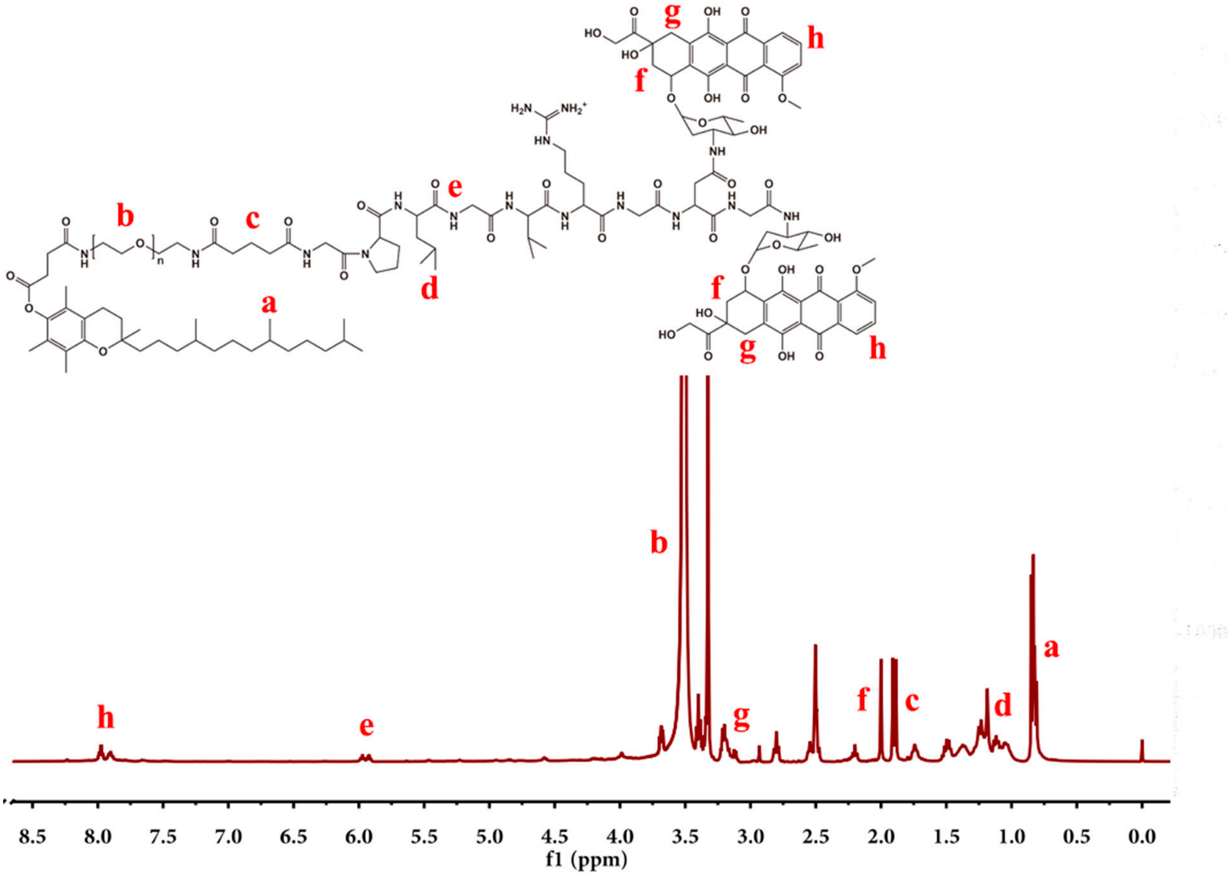

Figure S1. ^{1}H NMR spectrum of TPGS$_{3350}$-GPLGVRGDG-DOX.
